# Association of the Colleges of Dentistry

**Published:** 1867-01

**Authors:** 


### Proceedings of Societies.

### ASSOCIATION OF THE COLLEGES OF DENTISTRY.

During the meeting of the American Dental Association at Boston in August last, a conference was had by the representatives there present of the various Dental Colleges in the United States.

A free expression of opinion was given by those present upon the subject of dental education, particularly with reference to the preparation of those seeking to enter the ranks of the profession. All concurred in the opinion that much more than has yet been attained in this direction is desirable. This, in connection with the demand of the profession in reference to this matter, would seem to indicate that a concurrent advance movement should be made by our Colleges.

Harmony and uniformity in any advance movement was conceded by all to be desirable. Suggestions were made on many points, especially upon a basis for future action.

A preamble and constitution for an organization was presented, which was referred for future action, after which it was agreed to meet in Philadelphia at the call of the chairman. The Conference adjourned.

According to previous notice, the faculties of the Dental Colleges of the United States, met at the lecture room at the Philadelphia Dental College, in Philadelphia, at 10 A. M. October 17, 1866.

The different Colleges were represented as follows:

Baltimore Dental College, F. J. S. Gorgas.

Pennsylvania Dental College, T. L. Buckingham, Geo. T. Barker, E. Wildman, W. S. Forbes, J. Trueman.

Ohio Dental College, J. Taft.

Philadelphia Dental College, J. H. McQuillen, C. A. Kingsbury, J. F. Flagg, J. Garretson, A. Leeds, Thomas Wardle.

New York College of Dentistry, E. Parmley, N. W. Kingsley, W. H. Dwinelle.

Dr. Parmley was chosen chairman *pro tem.*, and J. Taft Secretary.

On motion of Dr. Buckingham, a committee consisting of one from each faculty was appointed, to nominate officers and prepare business for this meeting.

COMMITTEE.—T. L. Buckingham, J. H. McQuillen, F. J. S. Gorgas, W. H. Dwinelle, J. Taft.

The Committee having retired after due consultation, presented a partial report, which embraced a draft of a preamble and constitution for a permanent organization. The report being accepted, the constitution was read article by article, discussed, amended and adopted.

PREAMBLE.

WHEREAS we recognize the necessity for further effort for the advancement and elevation of our profession, and for a higher standard of education and professional attainments: therefore

*Resolved*, That we do form ourselves into an Association

Constitution of the Association of the Colleges of Dentists for the accomplishment of the above object, with the following regulations for our government.

ARTICLE I.

This organization shall be styled THE ASSOCIATION OF THE COLLEGES OF DENTISTRY, and shall be composed of the Faculties of the Dental Colleges subscribing to this Constitution.

ARTICLE II.

The duty of this organization shall be to confer together upon such means, and to suggest such measures to the various Colleges, as may lead to a concert of action in the furtherance of these objects.

ARTICLE III.

The officers of this Association shall be a President, Vice-President, Recording Secretary, Corresponding Secretary, and Treasurer.

ARTICLE IV.

The vote on all ordinary questions may be decided by the individual members of the Association present at the meetings; but the determination of any question of importance shall only be by a vote of the Colleges belonging to this organization. Each College being entitled to but one vote, and in case of a tie, the matter shall be referred back to the respective Faculties for decision; the Professors in the Didactic Course of each College being entitled to vote, and the majority shall decide.

ARTICLE V.

This Constitution may be amended or altered, by notice being given one year in advance to all the Faculties; two-thirds of the Colleges being necessary to effect such change.

It was, as a whole, fully discussed by Profs. Garretson, Kingsley, Buckingham and Flagg, after which it was unanimously adopted.

The Executive Committee now presented nominees for the various officers of the permanent organization.

The election of officers was now declared in order. Upon balloting, the following persons were found to be elected.

President.—E. Parmley, of New York.

Vice-President.—F. J. S. Gorgas, of Baltimore.

Rec. Secretary.—J. Taft, of Cincinnati.

Cor. Secretary.—J. H. McQuillen, of Philadelphia.

Treasurer.—Geo. T. Barker, of Philadelphia.

Adjourned to meet at the lecture room of the Pennsylvania Dental College at 4 o'clock P. M.

AFTERNOON SESSION.

College Association met at the time and place designated by the adjournment. Minutes of the morning session were read and approved.

On motion,

*Resolved*, That a Committee of three be appointed to transcribe this Constitution into a book, to be procured for the purpose. Also, to draft a code of by-laws, to be presented to the next meeting of the Association.

COMMITTEE.—J. Taft, N. W. Kingsley, J. H. McQuillen.

The Committee was requested to furnish each of the Faculties with a draft of the proposed by-laws, one month before the next meeting.

On motion of Dr. Gorgas,

*Resolved*, That the rule of our Colleges, allowing one session in a medical College to be considered equivalent to one course in a dental College, be abolished.

On motion of Dr. Kingsbury,

*Resolved*, That two years of pupilage with a reputable dental practitioner, inclusive of two full courses of lectures in a dental College, be required to entitle the candidate to an examination for graduation as D. D. S.

On motion of E. Wildman,

*Resolved*, That a graduate of a respectable medical College who has been under the pupilage of a reputable dentist for one year, and shall have attended one full course of lectures in a dental College, shall be entitled to examination for graduation.

*Resolved*, That seven years practice be regarded as equivalent to one course of lectures; the applicant shall pass a satisfactory preliminary examination before entering a dental College.

*Resolved*, That the regular term of instruction in the dental Colleges, be five months instead of four. (Laid on the table.)

*Resolved*, That students shall not be received later than the 20th of November; this to take effect the present year. All other regulations as agreed upon above, to take effect at the beginning of the session of 1867–8.

*Resolved*, That the tuition fees of the Colleges be for the first course $120, and for the second, $115. (Laid on the table.)

It was requested that the minutes of these meetings be published in the dental Journals.

The Association adjourned to meet in Philadelphia on the third Wednesday of March, 1867. J. TAFT,
*Secretary.*

